# Male Songbird Indicates Body Size with Low-Pitched Advertising Songs

**DOI:** 10.1371/journal.pone.0056717

**Published:** 2013-02-20

**Authors:** Michelle L. Hall, Sjouke A. Kingma, Anne Peters

**Affiliations:** 1 Vogelwarte Radolfzell, Max Planck Institute for Ornithology, Radolfzell, Germany; 2 Department of Zoology, University of Melbourne, Melbourne, Victoria, Australia; 3 School of Biological Sciences, University of East Anglia, Norwich, United Kingdom; 4 School of Biological Sciences, Monash University, Clayton, Victoria, Australia; University of Massachusetts, United States of America

## Abstract

Body size is a key sexually selected trait in many animal species. If size imposes a physical limit on the production of loud low-frequency sounds, then low-pitched vocalisations could act as reliable signals of body size. However, the central prediction of this hypothesis – that the pitch of vocalisations decreases with size among competing individuals – has limited support in songbirds. One reason could be that only the lowest-frequency components of vocalisations are constrained, and this may go unnoticed when vocal ranges are large. Additionally, the constraint may only be apparent in contexts when individuals are indeed advertising their size. Here we explicitly consider signal diversity and performance limits to demonstrate that body size limits song frequency in an advertising context in a songbird. We show that in purple-crowned fairy-wrens, *Malurus coronatus coronatus*, larger males sing lower-pitched low-frequency advertising songs. The lower frequency bound of all advertising song types also has a significant negative relationship with body size. However, the average frequency of all their advertising songs is unrelated to body size. This comparison of different approaches to the analysis demonstrates how a negative relationship between body size and song frequency can be obscured by failing to consider signal design and the concept of performance limits. Since these considerations will be important in any complex communication system, our results imply that body size constraints on low-frequency vocalisations could be more widespread than is currently recognised.

## Introduction

Signal honesty is fundamental for the evolutionary maintenance of communication. Many sexually selected signals are reliable because of physical or physiological constraints on signal production (‘index signals’) [1]. Body size is a key sexually selected trait [2], and the limit that size imposes on producing loud low-frequency sounds is a textbook example of an honesty-enforcing constraint [3]. The negative relationship between body size and call frequency has strong theoretical support [4], [5], as well as empirical support from comparative analyses across animal taxa [4], [5] and across species within taxa such as birds [6], [7] and primates [8]. This comparative empirical support has been demonstrated despite considerable differences between taxa and species in selection pressures, metabolic rates, and sound-producing mechanisms (for example, external in insects, internal in birds and mammals). However, the critical prediction for the hypothesis that size enforces signal honesty is not that this relationship exists between species (or even within a species between populations or the sexes), but rather that there is a negative relationship between vocalisation frequency and size among competing individuals.

Empirical support for a within-sex relationship between size and call frequency is limited largely to a single taxon with relatively simple calls. In anurans, the dominant frequency of advertising or aggressive calls is negatively correlated with body size in many species (reviewed in [9]). However, fundamental frequency rarely correlates with size in mammals [10], instead formant dispersion more commonly indicates size [11], [12]. For the complex vocalisations of songbirds, empirical support is rare (reviewed in [13], [14]) despite detailed study [15], and limited to a single example: larger male barn swallows sing the rattle syllable of their songs at lower frequencies [16]. Other studies have demonstrated frequency variation associated with size differences between populations [17], [18], likely due to population differences in habitat or selection pressures [18]. Within a population, competing individuals usually have similar hormonal profiles, metabolic rates, and sound-producing mechanisms (for an unusual exception in a grasshopper, see [19]), and are subject to similar selection pressures, so these factors are unlikely to obscure a relationship between body size and vocalisation frequency. However, the relationship could be obscured by vocal complexity, when frequency varies in vocal repertoires or with context (for examples of contextual differences in anurans, birds, and mammals, see [20]–[22]).

Songbirds display particularly complex, learned vocalisations that are sexually-selected and can be frequency modulated both stereotypically in repertoires of song types and dynamically with context [23]. Since frequency ranges are usually larger than those expected due to within-sex variation in size, this vocal complexity could obscure a relationship between body size and frequency [13], [14]. However, theory predicts that body size limits the production of loud, low-frequency sounds [3], so it is only the lower limit of the full frequency range that should be constrained by size. Likewise, in repertoires of divergent song types, size should constrain only low-frequency song types. If repertoire diversity is partly a consequence of different signals being designed to communicate different messages [24], [25], then only the low-frequency subset of song types may indicate size. Finally, if frequency varies dynamically with context, a size-frequency relationship may only be apparent in contexts where size is being advertised [21].

We tested whether song frequency is an index signal of body size in an oscine passerine, the purple-crowned fairy-wren (*Malurus coronatus coronatus*), by restricting the analysis to songs given in an advertising context, and explicitly considering signal design and lower frequency limits. We focused on a male advertising song that is analogous to the trill song that males of other *Malurus* species give in response to predator calls (also called ‘Type II’ song) [26]–[28]. These songs are thought to be directed at conspecifics and sexually selected because they are distinct from the alarm calls given by both sexes, are sung only by males (even though females also sing in this genus), because females tend to respond more strongly to these songs when they follow predator calls than when they are sung solo, and because trill length is related to male age, which predicts extra-pair mating success [26]–[28]. We elicited trill songs experimentally using playback of predator calls, rather than recording natural trill songs in the dawn chorus (when they are sung alongside ‘Type I’ songs), to give greater control of song context. We predicted that low-frequency trill song types in particular would show a relationship between frequency and male size. We also predicted that the *lower limit* of the relationship between body size and the song frequency of all trill song types would have a negative slope. However, if different song types in a repertoire are designed to communicate different information (‘multiple messages’), we expected there might be no relationship between male body size and the average frequency of all their trill song types.

## Materials and Methods

### Ethics statement

All work was carried out according to relevant national and international guidelines, and under the following permits: Australian Bird and Bat Banding licence 2073, WA Department of Environment and Conservation permits SF007544 and BB002798, and Max Planck Institute for Ornithology Ethics permit.

### Study species

We studied a colour-banded population of purple-crowned fairy-wrens at Mornington Wildlife Sanctuary, Australia, that included approximately 45 to 65 territories from 2005 to 2010 (for details see [29], [30]). Purple-crowned fairy-wrens are long-lived riparian specialists that breed cooperatively [31], [32]. Groups live in all-purpose territories that the breeding pair defends year-round using repertoires of songs that are sung solo or together in duets [29]. In addition to these songs, males sometimes sing a short trilled song composed of rapidly repeated syllables that is the focus of this study. We captured birds with mistnets for banding and morphometric measurements, measuring tarsus length (±0.1 mm) twice at each capture, and using the average of all measurements for each individual as a measure of body size. The species is the largest member of its genus [33], and males are larger than females (in this population, for 90 females and 84 males respectively, mass = 10.49±0.06 g and 11.16±0.06 g, t = −7.73, *p*<0.001; tarsus = 23.52±0.07 mm and 24.47±0.07 mm, t = −9.443, *p*<0.001). We used tarsus length as our measure of body size because it is a measure that predicts female choice in some birds [34], [35], and because it was related to call frequency in other studies [21], [23]. Furthermore, tarsus length is constant in adults, and multiple measurements of individuals over the course of our study were highly repeatable (adjusted R^2^ = 0.92, effect of individual identity in one-way ANOVA F_371,1426_ = 53.8, *p*<0.0001). We did not use mass as a measure of body size because males were not captured at the same time as their songs were recorded and mass varies over time.

### Song playback and recording

We elicited advertising songs using playback to the breeding male of each group (helper males sang trill songs very rarely). Butcherbirds (*Cracticus* spp) are predators known to elicit trill (‘Type II’) songs from other fairy-wren species [26], and brush cuckoos (*Cacomantis variolosus*) are brood parasites whose ‘police whistle’ call is highly effective in eliciting trill songs from purple-crowned fairy-wrens (MLH pers obs). As playback stimuli, we used recordings of a pied butcherbird (*Cracticus nigrogularis*) from the BOCA Australian Birdsong CD series, a brush cuckoo ‘police whistle’ call recorded onsite using a Sennheiser ME67 microphone and a Marantz PMD660 digital recorder, and a synthesised version of the brush cuckoo call (3300 Hz tone of 500 msec duration, with 18 Hz sin frequency modulation and 35 Hz modulation range) created in the program SYRINX (John Burt, www.syrinxpc.com). Stimuli were transferred to an iPod in uncompressed .wav format, and broadcast via a Sony SRS-A27 speaker at approximately natural amplitudes. We repeated playback interactively to avoid overlap and occasionally switched playback type to minimise habituation, with the goal of eliciting multiple high quality recordings of trill songs from each male for acoustic analysis. We recorded elicited songs with a Sennheiser ME67 microphone and a Marantz PMD660 digital recorder at a sampling frequency of 44.1 kHz, usually from within 5 or 10 m of the singer to maximise recording quality.

### Acoustic analysis

**Figure 1:**
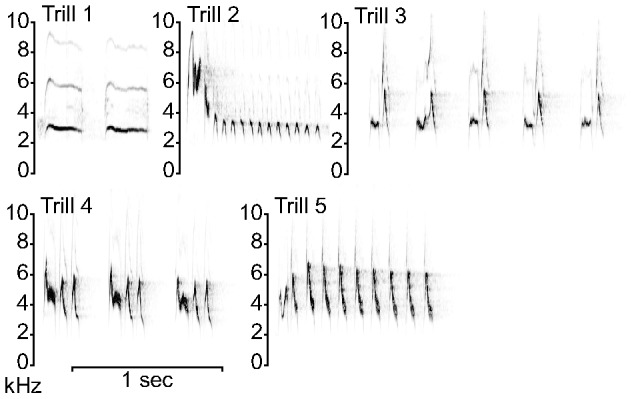
Signal designs of trill songs. Sonagrams illustrating the five common trill song types recorded in the population. In these examples, Trill 2 and Trill 5 contain introductory syllables preceding the main trill phrase. Go to Supporting Information Audio S1, S2, S3, S4, S5 to listen to the trill songs illustrated here.

We obtained high quality recordings of 417 songs from 45 males (9.2±1.0 songs on 2.0±0.2 days per male). Songs consisted of a trill phrase (multiple repetitions of the same syllable) that was sometimes preceded by introductory syllables (Fig. 1). We grouped songs into types based on the acoustic structure of the repeated trill syllables (Fig. 1 shows the five common trill types, with corresponding audio files in Supplementary Information; three other types each sung by only one male are not illustrated and were excluded from further analysis). The sampling protocol was not designed to estimate individual repertoire sizes, but we obtained measurements from multiple renditions of a single type for 35 males (3 to 19 songs recorded), two types for 7 males (4 to 28 songs recorded), and three types for 3 males (17 to 31 songs recorded).

For acoustic analysis, we used spectrograms generated in Avisoft-SASLab Pro v4.39 (Avisoft Bioacoustics, Berlin, Germany). Spectrogram parameters gave a frequency resolution of 86 Hz and a temporal resolution of 2.9 ms (Hamming window, FFT = 512, overlap = 75%). We measured the peak frequency (frequency at peak amplitude) of each syllable in the main trill phrase using Avisoft's ‘magic reticule cursor’, which identifies the frequency with maximum energy in the spectra. Where syllables contained more than one element (Trill 3 and 4), we used the lowest peak frequency. We checked each detection visually to ensure the automated cursor had identified the peak frequency of the syllable itself, rather than background noise or the playback stimulus, in which case we either filtered out the noise or excluded that syllable from the analysis. We calculated song frequency as the average of the lowest peak frequency of each syllable in the main trill phrase. We used lowest peak frequency [23] rather than minimum frequency because high-amplitude low-frequency sounds are difficult to produce, since increasing sound amplitude tends to increase sound frequency (raising the pressure of air moving past sound-producing membranes passively increases oscillation rate). High-amplitude low-frequency sounds are also more likely to be detected by receivers than low-amplitude minimum frequencies, so we expect signals designed to advertise body size to have low peak frequencies.

### Statistical analysis

For each male, we calculated the mean song frequency for each trill type, and analysed these in a mixed model including male identity as a random effect, and trill type, tarsus length, and their interaction as fixed effects. We then demonstrate three approaches to testing for a relationship between body size and trill frequency using linear regression. (i) Signal design approach: using low-frequency trill types only (Trills 1, 2, and 3, see Results), we computed an average frequency for each male, and tested tarsus length as a predictor of frequency. (ii) Lower bound approach: using the full sample of songs, we tested for a negative slope in the lower limit of the relationship between frequency and body size, following the standard approach for assessing performance limits [23], [36]. In brief, we described the lower bound of the distribution of points by identifying the song with the lowest frequency in each size category (tarsus lengths binned into 0.25 mm intervals up to 25.25 mm, pooling the single larger male into the last bin to avoid including non-minimal events) [37], and fitted a regression line through this subset of points. (iii) We computed an average frequency for each male from the full sample of songs, and tested whether tarsus length predicts frequency.

We used Genstat 14^th^ edition for computation, and present means with standard errors. Data are available on request from MLH.

## Results

Songs elicited by playback spanned a range of signal designs (Fig. 1) that differed in lowest peak frequency (Table 1, Mixed Model trill type main effect: F_4,41.4_ = 54.90, *p*<0.001). Tarsus length had a significant effect on trill frequency in interaction with trill type (Tarsus length*Trill type interaction: F_4,42.6_ = 2.62, *p* = 0.048), but not overall (main effect: F_1,33.5_ = 0.01, *p* = 0.91). Longer tarsus lengths were only associated with lower frequency in low-frequency trill types (see predicted mean frequencies and effect sizes in Table 1 for Trills 1, 2 and 3 versus 4 and 5).

**Table 1:** Only low-frequency trill types (*) are negatively related to tarsus length.

Trill type	Lowest peak frequency (kHz)	Tarsus*trill type	Sample size
	(predicted means)	(effect sizes)	(males, songs)
1*	3.50	−0.71	9, 68
2*	3.65	−0.56	4, 19
3*	3.30	−0.90	4, 29
4	4.12	−0.09	4, 23
5	5.51	1.30	35, 272

The table shows Mixed Model predictions for each trill type (column 1), of mean frequency (column 2; average s.e.d. = 0.29) and the effect of tarsus on frequency (column 3; average s.e.d. = 0.48), as well as sample sizes (column 4, see Results for further details of Mixed Model output).

**Figure 2:**
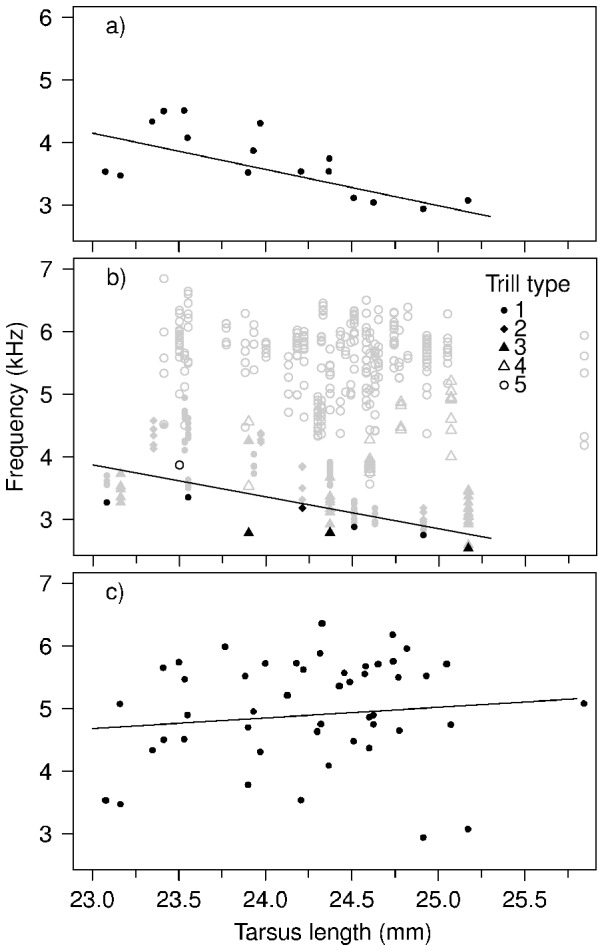
Body size limits low-frequency songs. a) Low-frequency trill song types (Trills 1, 2 and 3) were sung at lower frequencies by large than small males (regression line: y = −0.58x+17.49). Points represent mean peak frequency for each male, including Trills 1, 2 and 3. b) The lower bound of the relationship between frequency and body size had a negative slope (y = −0.51x+15.60, regression line fitted through black points, with minimum frequency in each tarsus length bin, see Methods). Each point represents a song. c) Tarsus length does not predict the mean frequency of all a male's trill song types (y = 0.17x+0.77). Points represent mean peak frequency for each male, including all trill types.

The signal design approach (i) showed that low-frequency trill types (Trills 1–3) were sung at significantly lower frequencies by large males than by small males (β = −0.58±0.17, *F*_1,13_ = 11.18, *p* = 0.005, Fig. 2a). The lower bound approach (useful in systems without discrete song ‘types’) (ii) showed that the relationship between tarsus length and frequency had a lower bound regression line with a significant negative slope (β = −0.46±0.14, *F*_1,7_ = 10.26, *p* = 0.02; Fig. 2b). However, disregarding signal design and performance limits and using the average frequency of all songs a male sang (approach iii) resulted in no apparent relationship between body size and frequency (β = 0.17±0.22, *F*_1,43_ = 0.65, *p* = 0.42, Fig. 2c).

## Discussion

We have shown that song frequency indicates body size in a songbird. The within-sex relationship between size and frequency predicted by this textbook example of a physical constraint ensuring signal honesty [3] has been demonstrated only once before in a songbird [16] despite several studies (reviewed in [13], [14]). Comparative studies [6], [7] fail to address the key prediction that there should be a negative relationship between vocalisation frequency and size among competing individuals for size to play a role in ensuring signal honesty. Our analysis suggests that the lack of support for this prediction in songbirds may be partly a result of failing to consider vocal diversity and focus on low-frequency limits of elaborate acoustic displays.

Body size is a sexually selected trait in some passerine birds [34], [35], but not all species experience selection to advertise body size vocally, and in fact females in some species prefer high-pitched song (reviewed in [14]). For example, among Icterids, the songs of polygynous species are close to the lower frequency limit imposed by body size, while other species in the family are not [23]. Furthermore, since some birds vary frequency dynamically to reduce the peak frequency of their songs during aggressive interactions [21], [23], peak frequency may only correlate with body size in contexts where advertising size is important [21]. The role that body size plays in purple-crowned fairy-wrens is currently unknown, but our findings from a class of songs given in an advertising context [26] indicate that this warrants further investigation.

There were considerable differences in the design of trill songs sung in the same context (Fig. 1, Table 1). Only three of five trill types minimised frequency (Trills 1, 2, and 3), and the negative relationship between body size and frequency was only detected in these low-frequency song types (effect sizes in Table 1, regression line in Fig. 2a). The lack of an overall size effect (main tarsus effect in Mixed Model, regression line in Fig. 2c) suggests that different trill types may be designed to convey different information. For example, although the design of Trill 5 does not minimise frequency, it may convey other information to receivers since its design simultaneously maximises syllable rate and bandwidth (Fig. 1), a song performance measure that is sexually selected in some species [36], [37]. This is the most common trill type, and its signal function warrants further investigation. If song types given in the same context are not functionally equivalent, this suggests that the evolution of vocal complexity in songbirds may be driven by selection to communicate multiple messages, rather than merely to maximise diversity [38]. Thus, future studies should consider signal structure when investigating the honesty of the information signals convey.

The mechanism underlying the negative relationship between body size and song frequency in purple-crowned fairy-wrens could be direct or indirect. The physical constraint hypothesis suggests a direct limit imposed by body size on low-frequency songs because low-frequency sounds require longer and larger medial syringeal labia [39] and expansion of the upper end of the oesophagus to a large size [40], and their size is limited because they are internal organs. Nevertheless, the soft tissues involved might reduce the constraint [41], though body size also influences song frequency via metabolic rate [5]. An alternative hypothesis is that, since developmental stress can affect sexually selected traits [42], it could cause an indirect relationship between body size and song frequency if developmental stress affected both body size and vocal system development. In that case, smaller males might be unable to sing loud low-pitched songs as a result of stress during development rather than a size constraint. Further experimental work is necessary to distinguish these hypotheses, and to determine how body size influences outcomes of competition in purple-crowned fairy-wrens.

## Supporting Information

Audio S1 **Audio for Trill 1 example illustrated in** **Figure 1** **.**
(WAV)

Audio S2 **Audio for Trill 2 example illustrated in** **Figure 1** **.**
(WAV)

Audio S3 **Audio for Trill 3 example illustrated in** **Figure 1** **.**
(WAV)

Audio S4 **Audio for Trill 4 example illustrated in** **Figure 1** **.**
(WAV)

Audio S5 **Audio for Trill 5 example illustrated in** **Figure 1** **.**
(WAV)

## References

[1] Maynard Smith J, Harper DGC (1995) Animal signals: models and terminology. Journal of Theoretical Biology 177: 305–311.

[2] Hunt J, Breuker CJ, Sadowski JA, Moore AJ (2009) Male-male competition, female mate choice and their interaction: determining total sexual selection. Journal of Evolutionary Biology 22: 13–26. 19120810 10.1111/j.1420-9101.2008.01633.x

[3] Bradbury JW, Vehrencamp SL (1998) Principles of animal communication. Sunderland, Massachusetts: Sinauer Associates Inc.

[4] Fletcher NH (2004) A simple frequency-scaling rule for animal communication. Journal of the Acoustical Society of America 115: 2334–2338. 15139646 10.1121/1.1694997

[5] Gillooly JF, Ophir AG (2010) The energetic basis of acoustic communication. Proceedings of the Royal Society of London Series B: Biological Sciences 277: 1325–1331. 20053641 10.1098/rspb.2009.2134 PMC2871947

[6] Ryan MJ, Brenowitz EA (1985) The role of body size, phylogeny, and ambient noise in the evolution of bird song. The American Naturalist 126: 87–100.

[7] Martin JP, Doucet SM, Knox RC, Mennill DJ (2011) Body size correlates negatively with the frequency of distress calls and songs of Neotropical birds. Journal of Field Ornithology 82: 259–268.

[8] Hauser MD (1993) The evolution of nonhuman primate vocalizations - effects of phylogeny, body weight, and social context. The American Naturalist 142: 528–542. 10.1086/285553 19425989

[9] Searcy WA, Nowicki S (2005) The evolution of animal communication: reliability and deception in signaling systems. Princeton: Princeton University Press.

[10] Evans S, Neave N, Wakelin D (2006) Relationships between vocal characteristics and body size and shape in human males: An evolutionary explanation for a deep male voice. Biological Psychology 72: 160–163. 16280195 10.1016/j.biopsycho.2005.09.003

[11] Reby D, McComb K (2003) Anatomical constraints generate honesty: acoustic cues to age and weight in the roars of red deer stags. Animal Behaviour 65: 519–530.

[12] Taylor AM, Reby D, McComb K (2010) Size communication in domestic dog, *Canis familiaris*, growls. Animal Behaviour 79: 205–210.

[13] Patel R, Mulder RA, Cardoso GC (2010) What makes vocalisation frequency an unreliable signal of body size in birds? A study on black swans. Ethology 116: 554–563.

[14] Cardoso GC (2012) Paradoxical calls: the opposite signaling role of sound frequency across bird species. Behavioral Ecology 23: 237–241.

[15] Cardoso GC, Mamede AT, Atwell JW, Mota PG, Ketterson ED, et al (2008) Song frequency does not reflect differences in body size among males in two oscine species. Ethology 114: 1084–1093.

[16] Galeotti P, Saino N, Sacchi R, Moller AP (1997) Song correlates with social context, testosterone and body condition in male barn swallows. Animal Behaviour 53: 687–700.

[17] Handford P, Lougheed SC (1991) Variation in duration and frequency characters in the song of the rufous-collared sparrow, *Zonotrichia capensis*, with respect to babitat, trill dialects and body size. Condor 93: 644–658.

[18] Irwin DE, Thimgan MP, Irwin JH (2008) Call divergence is correlated with geographic and genetic distance in greenish warblers (*Phylloscopus trochiloides*): a strong role for stochasticity in signal evolution? Journal of Evolutionary Biology 21: 435–448. 18205774 10.1111/j.1420-9101.2007.01499.x

[19] Donelson NC, van Staaden MJ (2005) Alternate tactics in male bladder grasshoppers *Bullacris membracioides* (Orthoptera : Pneumoridae). Behaviour 142: 761–778.

[20] Yin S, McCowan B (2004) Barking in domestic dogs: context specificity and individual identification. Animal Behaviour 68: 343–355.

[21] Geberzahn N, Goymann W, Muck C, ten Cate C (2009) Females alter their song when challenged in a sex-role reversed bird species. Behavioral Ecology and Sociobiology 64: 193–204. 19946649 10.1007/s00265-009-0836-0 PMC2779343

[22] Bee MA, Perrill SA, Owen PC (2000) Male green frogs lower the pitch of acoustic signals in defense of territories: a possible dishonest signal of size? Behavioral Ecology 11: 169–177.

[23] Price JJ, Earnshaw SM, Webster MS (2006) Montezuma oropendolas modify a component of song constrained by body size during vocal contests. Animal Behaviour 71: 799–807.

[24] Byers BE (1995) Song types, repertoires and song variability in a population of chestnut-sided warblers. Condor 97: 390–401.

[25] Vallet E, Kreutzer M (1995) Female canaries are sexually responsive to special song phrases. Animal Behaviour 49: 1603–1610.

[26] Greig EI, Pruett-Jones S (2010) Danger may enhance communication: predator calls alert females to male displays. Behavioral Ecology 21: 1360–1366.

[27] Dalziell AH, Cockburn A (2008) Dawn song in superb fairy-wrens: a bird that seeks extrapair copulations during the dawn chorus. Animal Behaviour 75: 489–500.

[28] Langmore NE, Mulder RA (1992) A novel context for bird song - predator calls prompt male singing in the kleptogamous superb fairy-wren, *Malurus cyaneus* . Ethology 90: 143–153.

[29] Hall ML, Peters A (2008) Coordination between the sexes for territorial defence in a duetting fairy-wren. Animal Behaviour 76: 65–73.

[30] Hall ML, Peters A (2009) Do male paternity guards ensure female fidelity in a duetting fairy-wren? Behavioral Ecology 20: 222–228.

[31] Kingma SA, Hall ML, Arriero E, Peters A (2010) Multiple benefits of cooperative breeding in purple-crowned fairy-wrens: a consequence of fidelity? Journal of Animal Ecology 79: 757–768. 20443991 10.1111/j.1365-2656.2010.01697.x

[32] Kingma SA, Hall ML, Peters A (2011) Multiple benefits drive helping behavior in a cooperatively breeding bird: an integrated analysis. The American Naturalist 177: 486–495. 10.1086/658989 21460570

[33] Higgins PJ, Peter JM, Steele WK, editors (2001) Handbook of Australian, New Zealand and Antarctic birds, Vol. 5. Oxford: Oxford University Press.

[34] Kempenaers B, Verheyen GR, Vandenbroeck M, Burke T, Vanbroeckhoven C, et al (1992) Extra-pair paternity results from female preference for high-quality males in the blue tit. Nature 357: 494–496.

[35] Møller AP, Ninni P (1998) Sperm competition and sexual selection - a meta-analysis of paternity studies of birds. Behavioral Ecology and Sociobiology 43: 345–358.

[36] Podos J (1997) A performance constraint on the evolution of trilled vocalizations in a songbird family (Passeriformes: Emberizidae). Evolution 51: 537–551. 28565357 10.1111/j.1558-5646.1997.tb02441.x

[37] Illes AE, Hall ML, Vehrencamp SL (2006) Vocal performance influences male receiver response in the banded wren. Proceedings of the Royal Society of London Series B: Biological Sciences 273: 1907–1912. 16822751 10.1098/rspb.2006.3535 PMC1634778

[38] Byers BE, Kroodsma DE (2009) Female mate choice and songbird song repertoires. Animal Behaviour 77: 13–22.

[39] Riede T, Goller F (2010) Functional morphology of the sound-generating labia in the syrinx of two songbird species. Journal of Anatomy 216: 23–36. 19900184 10.1111/j.1469-7580.2009.01161.x PMC2807973

[40] Riede T, Suthers RA, Fletcher NH, Blevins WE (2006) Songbirds tune their vocal tract to the fundamental frequency of their song. Proceedings of the National Academy of Sciences of the United States of America 103: 5543–5548. 16567614 10.1073/pnas.0601262103 PMC1459391

[41] Fitch WT (2000) Skull dimensions in relation to body size in nonhuman mammals: The causal bases for acoustic allometry. Zoology-Analysis of Complex Systems 103: 40–58.

[42] Spencer KA, MacDougall-Shackleton SA (2011) Indicators of development as sexually selected traits: the developmental stress hypothesis in context. Behavioral Ecology 22: 1–9.

